# A medical student’s perspective on motivating medical students for social accountability

**DOI:** 10.30476/jamp.2020.87235.1300

**Published:** 2021-10

**Authors:** PRINCY BIJU, ANNABEL MENSAH-DJAN, SANA HASHEMI, IQRAH ASLAM, RAMEEZ SHAHZAD

**Affiliations:** 1 GKT School of Medical Education, King's College London, London, United Kingdom; 2 Kings College London, Guy's Campus, Great Maze Pond, London, United Kingdom

## Dear Editor

We found the article by Mohammadi et al. ( 1 ), on medical students’ perspectives and motivation for social accountability to be very insightful and thought-provoking. We would like to, however, provide some insights that we think may be pertinent. Firstly, we believe that although grounded theory provided greater validity to this research, the use of snowball sampling limited the variability of sample. The research by Ilker et al. ( 2 ) proposed that conclusions from snowball sampling do not represent the entire population unless there is a very large sample size. Kelly et al. ( 3 ) also propose that snowball sampling invites bias into the research as most people form friendships with those that have similar interests to themselves. Therefore, initial participants are more likely to nominate people that they know, which could result in participants that share similar traits and characteristics. This could mean that the sample obtained only contained a small subgroup of the entire population; therefore, results are not the representative of all and cannot be generalized. Furthermore, Shaghagi et al. ( 4 ) propose that snowball sampling is an effective method to target ‘hard-to-reach’ populations. This does not apply to this research as medical students cannot be classed as ‘hard-to-reach’ because they could have been identified and randomly selected from university databases. We believe that a stratified random sampling method would have been more appropriate to be used in this research as it would have ensured a higher degree of representation of all groups, thus helping to strengthen the external validity. 

In addition, we found that factors such as ethnicity, age and gender could have an impact on motivation. Marley et al. ( 5 ) found that younger students are less motivated for medicine than older ones. Furthermore, research by Leyerzapf et al. ( 6 ) found that students from ethnic minority backgrounds reported experiencing ethnicity-related barriers, and this, therefore, affected their performance and motivation for studying medicine. Taking this into account, these additional factors could have been explored to provide a better understanding of social accountability in medical students.

Finally, it is evident from previous research that there are confounding variables that need to be explored prior to concluding that creating purposeful beliefs and behaviour is the most important aspect for the development of students’ motivation for social accountability. We believe that stratified random sampling would have enabled the findings to be more representative rather than the use of a snowball sampling method. Moreover, factors such as ethnicity, age and gender could have an impact on motivation; therefore, they would have been valuable factors to have been used in this research paper. 
